# Obesity Risk Was Associated with Alcohol Intake and Sleep Duration Among Korean Men: The 2016–2020 Korea National Health and Nutrition Examination Survey

**DOI:** 10.3390/nu16223950

**Published:** 2024-11-19

**Authors:** Sang Young Kim, Hyun Ja Kim

**Affiliations:** Department of Food and Nutrition, Gangneung-Wonju National University, 7 Jukheon-gil, Gangneung-si 25457, Republic of Korea; sangaustin@gmail.com

**Keywords:** obesity, alcohol, sleep, men, Korea National Health and Nutrition Examination Survey

## Abstract

Background/Objectives: Excessive alcohol drinking and short sleep duration could be problematic in terms of obesity. This research investigated the risk of obesity according to alcohol consumption and sleep duration, using data from the 2016–2020 Korea National Health and Nutrition Examination Survey (KNHANES). Methods: The health behavior survey on alcohol intake and sleep duration was conducted via self-reporting by participants. Height and weight were measured to calculate the body mass index (BMI), which was then used to assess obesity, defined as a BMI of ≥25 kg/m^2^. Among a total of 39,738 participants from the 2016–2020 KNHANES, this study finally included 8271 Korean men aged ≥30 years, with 3467 classified as obese and 4804 as controls. Results: Obesity risk was significantly increased with a short sleep duration of <7 h (vs. 7–<9 h, odds ratio [OR] = 1.28, 95% confidence interval [95% CI] = 1.15–1.43) or frequency of binge drinking of ≥1 time/week (vs. never, OR = 1.39, 95% CI = 1.17–1.65). Moreover, the risk of obesity was further increased to 1.52 (95% CI = 1.17–1.97) for those with both short sleep duration and frequent binge drinking. Conclusions: The risk of obesity was elevated for frequent binge alcohol drinking with short sleep duration in Korean men.

## 1. Introduction

Obesity has been a global public health concern and is a disease that needs to be treated. The prevalence of obesity in Korea is 39.0% for people aged ≥30 years; specifically, the prevalence is much higher in men (49.1%) than in women (27.9%), according to the 2022 Korea National Health and Nutrition Examination Survey (KNHANES) [1]. Obesity has been significantly linked to an elevated risk of many diseases, including hypertension, type 2 diabetes mellitus, cardiovascular disease, and cancer [2,3]. There have been many factors, such as diets and lifestyles, identified that can influence obesity [4]. In more detail, alcohol consumption has been a well-known risk factor for obesity, and one of the reasons is that a high amount of alcohol drinking can contribute to excess energy consumption [5]. This is because alcohol yields 7 kcal/g, which is higher than its carbohydrate and protein contents of 4 kcal/g, respectively [5]. In Korea, alcohol consumption is incomparably higher in men than in women (23.9 g/day and 5.8 g/day in 2016–2018, respectively) [6], and the rate of monthly binge drinking for men was 48.8% in 2022 [1]. Besides alcohol intake, short sleep duration has been recognized as another obesity risk factor. In a previous meta-analysis, short sleep duration was related to elevated obesity risk [7], as it is plausible that short sleep duration diminishes leptin hormone levels and increases ghrelin hormone levels [8]. These hormonal alterations increase the appetite and promote weight gain. Similar to short sleep duration, alcohol drinking can also change the effects of hormones such as leptin [9]. Furthermore, alcohol drinking was related to interfered sleep homeostasis, affecting sleep disturbances [10], and short sleep duration was even related to increased alcohol consumption [11]. Therefore, the combined effect of excessive alcohol drinking and short sleep duration may cause a greater risk of obesity than their respective effects; however, research on this topic is sparse in Korea.

Considering the high alcohol drinking and substantially increased prevalence of obesity among men in Korea, this study focused solely on men. The purpose of this research was to investigate whether obesity risk was related to alcohol consumption and sleep duration among Korean men aged ≥30 years using the 2016–2020 Korea National Health and Nutrition Examination Survey (KNHANES).

## 2. Materials and Methods

### 2.1. Study Design and Participants

This study was completed using data from the 2016–2020 KNHANES, representing the Korean population. The purpose of this cross-sectional survey administered by the Korea Disease Control and Prevention Agency (KDCA) was to examine the health and nutritional status of Koreans. The survey was initially conducted at 3- or 4-year intervals in 1998, 2001, and 2005, but data collection has been performed annually since 2007 to ensure timely data provision. A total of 39,738 individuals participated in the 2016–2020 KNHANES, and the target population of this research was 12,358 Korean men aged ≥30 years. Participants with cardiovascular disease (*n* = 843) and cancers (*n* = 528) were excluded since their obese status could have been affected by these diseases. Another reason for these exclusions was that their alcohol drinking habits could have changed accordingly. Additionally, participants with cirrhosis (*n* = 42) were also excluded for the same reason related to alcohol drinking. Next, those with available BMI data and 500 kcal ≤energy intake ≤5000 kcal were included. Finally, a total of 8271 participants were selected for this research, specifically, 3467 obese and 4804 controls. Written informed consent was received from each participant, and the survey procedure was approved by the KDCA Institutional Review Board (waived in 2016 and 2017, 2018-01-03-P-A, 2018-01-03-C-A, and 2018-01-03-2C-A).

### 2.2. Data Collection

Body mass index (BMI) was calculated as weight in kilograms divided by height in meters squared, based on height measured by a stadiometer (Seca, Hamburg, Germany) and weight measured by a GL-6000-20 scale (G-tech, Seoul, Republic of Korea) in a mobile examination vehicle. Obesity was defined as a BMI of ≥25 kg/m^2^, based on the criteria for the Asia-Pacific region (underweight as <18.5 kg/m^2^, normal range as 18.5–22.9 kg/m^2^, overweight as 23–24.9 kg/m^2^, and obese as ≥25 kg/m^2^), which was established according to the increased risk of co-morbidities for Asians [12]. Information on age, education, occupation, and monthly income was assessed by interviewing, and data on smoking and physical activity were obtained by self-reporting. The participants were classified into age groups of 30–39, 40–49, 50–64, and ≥65 years, and education levels of ≤elementary school, middle school, high school, and ≥college. Occupations were divided into professional and clerical, sales, service, agriculture, forestry, fishery, technician, and laborer, and unemployed (including housewives and students). The categories of monthly income level were low, middle-low, middle-high, or high; smoking status was never, past, or current; and physical activity was light, moderate (1–4 days of walking in the past week or walking < 30 min daily for ≥5 days in the past week), or vigorous (walking ≥30 min daily for ≥5 days in the past week). Total daily energy and nutrient intakes were estimated via a 24 h dietary recall survey issued by a trained dietitian at each participant’s home.

### 2.3. Alcohol Intake Assessment

Alcohol consumption was surveyed by the participants’ self-reports through the health behavior survey. There were three questions based on an individual’s alcohol drinking experience in the past year. A question about the frequency of binge drinking of ≥7 glasses/once was “how often do you drink 7 glasses (or about 5 cans of beer) or more from each glass, regardless of soju or liquor at a drinking occasion?” The responses were “never”, “<1 time/month”, “1 time/month”, “1 time/week”, and “almost every day”. Then, these responses were classified as never (non-drinkers), non-binge drinking, binge drinking of <1 time/week, or binge drinking of ≥1 time/week.

### 2.4. Sleep Duration Evaluation

The average daily sleep duration on weekdays and weekends was described by self-reporting via the health behavior survey. Specifically, for the 2016–2018 surveys, two questions were asked, as follows: “What time do you usually go to bed and wake up on weekdays (or working days)?” and “what time do you usually go to bed and wake up on weekends (or non-working days)?” The participants responded in an open-ended form with hours and minutes regarding their bedtime and wake-up time, respectively, then the responses were converted into hours of sleep duration. For the 2019–2020 surveys, the question of “how many hours a day do you usually sleep?” was asked. The participants answered in an open-ended manner in hours of their sleep duration on weekdays (or working days) and weekends (or non-working days), respectively. The total sleep duration was calculated by weighting 5 days for the weekdays and 2 days for the weekends. Finally, based on a previous systematic review and meta-analysis research [13], the sleep duration was classified as short sleep duration of <7 h, reference sleep duration of ≥7 to <9 h, and long sleep duration of ≥9 h. In stratified analyses, sleep duration was grouped as <7 h and ≥7 h.

### 2.5. Statistical Analyses

This research utilized SAS software, version 9.4 (SAS Institute; Cary, NC, USA), with PROC SURVEY. In order to represent the Korean population, sampling weights, strata, and clusters were applied to all analyses for each year from 2016 to 2020. These sampling weights were generated considering the complexity of the sample design, the non-response rate of the target population, and post-stratification. The results were demonstrated as mean ± standard error (SE) for continuous variables and percentages and (SE) for categorical variables. The Rao–Scott chi-square test or *t*-test was used to compare obese and control groups. Also, the Rao-Scott chi-square test or ANOVA was used to compare differences in the three groups by sleep duration or four groups by frequency of binge drinking. Multivariable logistic regression was performed to obtain odds ratios (ORs) and 95% confidence intervals (95% CIs) for the risk of obesity. And various confounding variables were controlled for in the model, including age (years, continuous), education (≤elementary school, middle school, high school, or ≥college), occupation (professional and clerical, sales, service, agriculture, forestry, fishery, technician, and laborer, or unemployed), income level (low, middle-low, middle-high, or high), smoking (never, past, or current), physical activity (light, moderate, or vigorous), total energy intake (kcal/day, continuous), fat intake (g/day, continuous), sodium intake (mg/day, continuous), and vitamin C intake (mg/day, continuous). Two-sided *p*-values< 0.05 were treated as denoting statistical significance.

## 3. Results

### 3.1. General Characteristics of Obese Cases and Controls

The general characteristics of obese cases and controls are shown in Table 1. The mean ages of the 3467 obese subjects and 4804 controls were 49.1 ± 0.3 years and 52.2 ± 0.3 years, respectively. The percentage of those with a high education level (≥college) was greater in the obese group (53.5%) compared to the control group (47.6%) (*p* < 0.001). Additionally, there were more professional and clerical occupations in the obese group (38.9%) than in the controls (33.4%) (*p* < 0.001). There were no significant differences between the obese and control groups in terms of smoking (*p* = 0.341) and physical activity (*p* = 0.245). But, the obese group showed a higher proportion of binge drinking more than or equal to one time/week (37.5%) compared to the control group (31.0%) (*p* < 0.001). Moreover, sleep duration was statistically different between obese individuals (7.0 ± 0.0 h) and controls (7.1 ± 0.0 h) (*p* < 0.001). Also, the total daily energy intake was 2309.7 ± 18.0 kcal in the obese subjects, which was higher than the 2262.8 ± 14.9 kcal in the controls (*p* = 0.036).

### 3.2. General Characteristics According to Sleep Duration

Table 2 shows the general characteristics according to sleep duration. Participants with a sleep duration of ≥9 h showed a higher average age (57.1 ± 0.8 years) than those with a sleep duration of <7 h (50.5 ± 0.3 years) and 7–<9 h (50.1 ± 0.3 years). For education and occupation, there were higher percentages of ≤elementary school (27.1%) and unemployed (36.6%) and lower percentages of ≥college (31.1%) and professional and clerical (17.8%) for participants with a sleep duration of ≥9 h compared to those with shorter sleep durations. In addition, participants with a sleep duration of ≥9 h presented a higher proportion of low income (32.4%) than those with a sleep duration of <7 h (24.6%) and 7–<9 h (23.4%). No significant differences were observed for smoking (*p* = 0.230), physical activity (*p* = 0.050), and total energy intake (*p* = 0.065).

### 3.3. General Characteristics According to Frequency of Binge Drinking

Table 3 demonstrates the general characteristics according to the frequency of binge drinking. The average age of participants with binge drinking of less than one time a week or more than or equal to one time a week was 48.0 ± 0.3 or 48.9 ± 0.3 years, which was lower than the ages of non-drinkers (56.8 ± 0.4 years) and non-bingedrinkers (54.2 ± 0.4 years). Participants with a binge drinking habit of less than one time a week reported the highest percentages of the education level of college (58.5%), professional and clerical occupations (43.2%), and high income (28.2%) than the other groups. For smoking, current smokers represented the highest proportion in the group with binge drinking of more than or equal to one time a week (48.2%) compared to the other groups. The lowest percentage of light physical activity was exhibited for participants with binge drinking of less than one time/week (17.7%). The proportion of sleeping less than 7 h was higher in the group with binge drinking of more than or equal to one time a week (44.2%) compared to the group with non-binge drinking (39.5%). The highest average of total energy intake was shown for participants with a binge drinking habit of more than or equal to one time a week (2470.5 ± 20.2 kcal).

### 3.4. Nutrient Intakes Related to Obesity According to Frequency of Binge Drinking

Table 4 represents nutrient intakes related to obesity by the frequency of binge drinking, adjusted for age. Participants who engaged in binge drinking more than or equal to one time a week consumed the highest intakes of total energy (2401.6 ± 20.2 kcal) and sodium (4169.8 ± 51.6 mg) and the lowest intakes of vitamin C (62.9 ± 2.2 mg), compared to the other groups. For fat intake, participants with binge drinking more than or equal to one time a week had a higher intake of 48.0 ± 0.8 g compared to non-drinkers.

### 3.5. Obesity Risk by Age Group, Sleep Duration, and Alcohol Intake

The results of the obesity risk by age group, sleep duration, and alcohol consumption are described in Table 5. The age group was not significantly related to the obesity risk. However, an increased risk of obesity was noted in participants with a short sleep duration of <7 h compared to participants with a relatively adequate sleep duration of 7–<9 h (OR = 1.28, 95% CI = 1.15–1.43). For the frequency of binge drinking, an elevated risk of obesity was observed for participants with binge drinking more than or equal to one time a week than for those who did not drink alcohol (OR = 1.39, 95% CI = 1.17–1.65).

### 3.6. Obesity Risk by Alcohol Intake as Frequency of Binge Drinking Stratified by Age Group and Sleep Duration

The risks of obesity regarding the frequency of binge drinking are stratified by age group and sleep duration in Table 6. Participants with a high frequency of binge drinking (more than or equal to one time a week) showed an increased risk of obesity than those without alcohol drinking in the 30–64 age group (OR = 1.38, 95% CI = 1.13–1.69), the ≥65 age group (OR = 1.38, 95% CI = 1.02–1.88), a sleep duration of <7 h (OR = 1.52, 95% CI = 1.17–1.97) and a sleep duration of ≥7 h (OR = 1.30, 95% CI = 1.03–1.63). Furthermore, a significantly higher risk of obesity was observed for those aged 30–64 years (OR = 1.45, 95% CI = 1.08–1.95) and ≥65 years (OR = 2.07, 95% CI = 1.28–3.33) who had a short sleep duration of <7 h with a high frequency of binge drinking compared to non-drinkers. Also, the obesity risk was elevated for participants in the 30–64 age group who had a sleep duration of ≥7 h and a high frequency of binge drinking compared to non-drinkers (OR = 1.32, 95% CI = 1.00–1.75). 

## 4. Discussion

In this research using data representing the Korean population, the key finding was that frequent binge drinking was significantly related to an increased risk of obesity for a short sleep duration of <7 h in men. 

Obesity has remained a serious disease worldwide because it is linked to the cause of many chronic diseases and disorders [14]. In more detail, obesity can directly elevate the morbidity and mortality of cardiovascular diseases due to several reasons, including changes in the heart structurally and functionally that can lead to heart failure [15]. Obesity can also contribute to oxidative stress and inflammation, which can eventually lead to diseases, such as cancers, due to DNA damage and cell metabolism issues [16]. In Korea, the prevalence of obesity dramatically increased over time in men aged ≥30 years, from 26.8% in 1998 to 49.1% in 2022, whereas there was no significant change in women (30.5% to 27.9%) [1]. This elevated trend is a serious problem that requires close attention, especially for men in Korea.

Among the many known risk factors for obesity, alcohol consumption, especially heavy drinking, has shown a significant effect on the increased risk of obesity [17]. Koreans consumed a high quantity of alcohol of 10.2 L/year in 2016, which was the second highest among Asian countries [18], and men generally consume a much higher amount of alcohol than women in Korea [6]. Worldwide, it was reported that the proportion of heavy episodic drinking among men was higher in Korea (49.9%) than in other countries, including South Africa (29.3%), Italy (39.7%), and the United States (44.7%) [18]. Unlike in the past, it is recommended not to drink alcohol to minimize the risks of health outcomes [19]. However, the drinking rate of less than or equal to one time a month for men was 67.6% in 2022 in Korea, which is still high [1].

In this study, men who engaged in frequent binge drinking exhibited an elevated risk of obesity. This might be because alcohol itself directly contributes to the total calories due to its high calorific value of 7 kcal/g [20]. Moreover, excessive alcohol drinking can bring increased energy intake through appetite-enhancing mechanisms, including the inhibitory effects of leptin, serotonin, and glucagon-like peptide-1, which can lead to overeating [9].

Additionally, Korea is known as a sleepless country, mainly because of Koreans’ work roles as people have rushed to achieve their goals due to rapid industrialization [21]. A previous study reported that the weekly sleep duration in Korea was 54.4 h, which was lower than that of Bulgaria (62.4 h), France (61.1 h), the United Kingdom (58.8 h), and the United States (58.5 h) [22]. Short sleep duration has been identified as a risk factor for obesity [7]. There are many possible mechanisms explaining how short sleep duration leads to obesity. For example, sleep deprivation can cause decreased physical activity due to tiredness, elevated chances for food consumption during the extra awake time, as well as increased appetite caused by the alteration of appetite-regulating hormones, specifically, diminishing leptin and elevating ghrelin [23]. The present study also found that short sleep duration was related to an increased obesity risk.

Furthermore, there has been a connection between sleep and alcohol intake. Alcohol consumption may help improve sleep initially, but high alcohol intake can eventually disrupt sleep as a result of the rapid development of tolerance to the sedative effects of drinking [24], as well as the impaired sleep homeostasis [10]. A previous study identified that binge drinking was significantly related to sleep problems, including trouble falling asleep and trouble staying asleep [25]. The results of the present study show that the frequent binge drinking group had the highest proportion of short sleep duration (<7 h) compared to the other groups. Furthermore, each short sleeping and alcohol-drinking episode can alter hormone levels, for example, leptin, which can result in an increased appetite [8,9]; thus, it is plausible that the simultaneous occurrence of a short sleep duration and alcohol consumption may significantly elevate the risk of obesity compared to their respective effects as a result of greater levels of hormonal changes.

Considering the results and respective effects of sleep duration and alcohol intake on obesity, the results of this study indicate that frequent binge drinking increased the risk of obesity in men with a short sleep duration of <7 h. In addition, the magnitudes of ORs were slightly greater for the older age group (≥65 years). Diminished muscle mass and increased fat mass can occur with age for several reasons, including changes in hormones and decreases in physical activity, which may naturally increase the risk of obesity [26]. It is also conceivable that older adults may be more vulnerable to the effects of alcohol intake and sleep deprivation than younger adults. Thus, in the older age group, a habit of binge drinking with a short sleep duration can be especially detrimental to health, eventually causing negative health consequences. Therefore, particular attention is needed in this regard.

Several strengths are shown in this research. To the best of our knowledge, this is the first research to investigate the combined effects of sleep duration and alcohol consumption on obesity in Korea. While previous studies focused on the relationship between two factors (e.g., obesity and alcohol intake, obesity and sleep, or alcohol intake and sleep), this study is novel in exploring their combined effects. Moreover, the KNHANES, which provides national data, represents the Korean population.

However, there are several limitations. First, it is challenging to establish cause-and-effect relationships between variables, as KNHANES bears a cross-sectional study design. Second, as sleep duration and binge drinking frequency data rely on self-reports, recall bias may be present. Third, the differences in the questions regarding sleep duration between the 2016–2018 and 2019–2020 surveys could be a limitation. Fourth, the participants may have underestimated the quantity or frequency of alcohol intake during the survey [27]. Fifth, more diseases associated with obesity could not be excluded from this study. For example, participants with hypothyroidism could not be excluded because thyroid examinations were not conducted and thus were not available for use in this research. Sixth, this research did not take into consideration the nutritional habits of the participants. Moreover, additional factors such as residential location, household composition, and work time pattern were not considered in this study, highlighting a need for future research to explore how these factors impact the findings to provide deeper insights. Lastly, even after controlling for numerous confounding factors in the analyses, residual confounding effects can still remain.

## 5. Conclusions

Using data from the 2016–2020 KNHANES, it was found that Korean men generally exhibited high rates of binge drinking and short sleep duration. Frequent binge drinking and short sleep duration were, respectively, associated with an increased risk of obesity. Furthermore, the risk of obesity was significantly increased for participants who experienced both short sleep duration and frequent binge drinking. This research hence suggests that it is necessary to sleep adequately and manage alcohol consumption as much as possible to reduce the risk of obesity. Further research considering the nutritional habits of individuals is needed to gain a more comprehensive understanding of their impact on this study’s outcomes.

## Figures and Tables

**Table 1:** General characteristics of obese cases and controls in Korean adult men aged ≥30 years, 2016 to 2020 Korea National Health and Nutrition Examination Survey.

	Obese (*n* = 3467)		Controls (*n* = 4804)		*p*-Values ^a^
Age (years, mean ± SE)	49.1	±0.3	52.2	±0.3	<0.001
Age (years, % (SE))					
30–39	27.5	(1.0)	21.3	(0.8)	<0.001
40–49	27.1	(0.9)	24.9	(0.8)	
50–64	32.9	(1.0)	34.3	(0.8)	
≥65	12.4	(0.6)	19.5	(0.6)	
Education (% (SE))					
≤Elementary school	7.9	(0.5)	11.8	(0.6)	<0.001
Middle school	8.0	(0.5)	9.5	(0.5)	
High school	30.7	(1.0)	31.1	(0.9)	
≥College	53.5	(1.2)	47.6	(1.1)	
Occupation (% (SE))					
Professional and clerical	38.9	(1.1)	33.4	(1.1)	<0.001
Sales, service, agriculture, forestry, fishery, technician, and laborer	45.7	(1.1)	46.6	(1.1)	
Unemployed	15.5	(0.7)	19.9	(0.7)	
Income level (% (SE))					
Low	24.1	(0.9)	25.0	(0.8)	0.357
Middle-low	25.7	(0.9)	24.4	(0.8)	
Middle-high	26.1	(0.8)	25.2	(0.7)	
High	24.1	(0.9)	25.4	(0.9)	
Smoking (% (SE))					
Never	22.6	(0.8)	21.3	(0.7)	0.341
Past	43.0	(1.0)	42.8	(0.8)	
Current	34.4	(0.9)	35.9	(0.9)	
Frequency of binge drinking (% (SE))					
Non-drinking	14.3	(0.7)	17.0	(0.7)	<0.001
Non-binge drinking	15.6	(0.7)	19.0	(0.6)	
<1 time/week	32.6	(1.0)	32.9	(0.9)	
≥1 time/week	37.5	(1.0)	31.0	(0.8)	
Physical activity (% (SE))					
Light	19.0	(0.8)	20.7	(0.7)	0.245
Moderate	53.3	(1.0)	51.6	(0.9)	
Vigorous	27.7	(0.9)	27.8	(0.8)	
Sleep duration (hours, mean ± SE)	7.0	±0.0	7.1	±0.0	<0.001
Total energy intake (kcal/d, mean ± SE)	2309.7	±18.0	2262.8	±14.9	0.036

^a^ *p*-values by Rao–Scott chi-square test for categorical variables or *t*-test for continuous variables.

**Table 2:** General characteristics according to sleep duration in Korean adult men aged ≥30 years, 2016 to 2020 Korea National Health and Nutrition Examination Survey.

	Sleep Duration						*p*-Values ^a^
	<7 h (*n* = 3432)		7–<9 h (*n* = 4056)		≥9 h (*n* = 783)		
Age (years, mean ± SE)	50.5	±0.3	50.1	±0.3	57.1	±0.8	<0.001
Age (years, % (SE))							
30–39	22.6	(0.9)	26.1	(0.9)	18.7	(1.8)	<0.001
40–49	27.3	(0.9)	25.7	(0.8)	19.6	(1.8)	
50–64	36.0	(1.0)	33.4	(0.9)	23.5	(1.8)	
≥65	14.2	(0.6)	14.9	(0.6)	38.2	(2.2)	
Education (% (SE))							
≤Elementary school	8.9	(0.5)	9.3	(0.5)	27.1	(2.1)	<0.001
Middle school	8.7	(0.6)	8.5	(0.5)	13.9	(1.9)	
High school	31.4	(1.0)	30.8	(1.0)	28.0	(2.3)	
≥College	51.1	(1.2)	51.4	(1.2)	31.1	(2.5)	
Occupation (% (SE))							
Professional and clerical	37.0	(1.2)	36.6	(1.1)	17.8	(2.0)	<0.001
Sales, service, agriculture, forestry, fishery, technician, and laborer	46.9	(1.1)	45.8	(1.1)	45.6	(2.7)	
Unemployed	16.1	(0.8)	17.6	(0.7)	36.6	(2.5)	
Income level (% (SE))							
Low	24.6	(1.0)	23.4	(0.8)	32.4	(2.0)	<0.001
Middle-low	24.1	(0.9)	25.2	(0.8)	28.2	(1.9)	
Middle-high	27.3	(0.9)	25.3	(0.8)	18.0	(1.5)	
High	24.0	(1.0)	26.1	(0.9)	21.5	(1.7)	
Smoking (% (SE))							
Never	22.1	(0.8)	22.1	(0.8)	18.5	(1.7)	0.230
Past	41.9	(1.0)	43.6	(0.9)	44.0	(2.2)	
Current	35.9	(1.0)	34.3	(0.9)	37.5	(2.2)	
Physical activity (% (SE))							
Light	19.2	(0.8)	19.9	(0.7)	26.0	(2.0)	0.050
Moderate	52.3	(1.0)	52.9	(0.9)	47.4	(2.6)	
Vigorous	28.5	(1.0)	27.2	(0.8)	26.6	(2.2)	
Total energy intake (kcal/day, mean ± SE)	2293.9	±17.9	2288.7	±15.6	2190.9	±41.6	0.065

^a^ *p*-values by Rao–Scott chi-square test for categorical variables or ANOVA for continuous variables.

**Table 3:** General characteristics according to frequency of binge drinking in Korean adult men aged ≥30 years, 2016 to 2020 Korea National Health and Nutrition Examination Survey.

	Never (*n* = 1477)		Non-binge Drinking (*n* = 1541)		Frequency of Binge Drinking ^a^				*p*-Values ^b^
					<1 Time/Week (*n* = 2521)		≥1 Time/Week (*n* = 2641)		
Age (years, mean ± SE)	56.8	±0.4	54.2	±0.4	48.0	±0.3	48.9	±0.3	<0.001
Age (years, % (SE))									
30–39	12.9	(1.2)	20.0	(1.3)	30.8	(1.2)	25.0	(1.1)	<0.001
40–49	20.0	(1.3)	21.5	(1.3)	26.5	(1.0)	30.2	(1.0)	
50–64	35.5	(1.6)	31.8	(1.5)	32.4	(1.1)	34.9	(1.1)	
≥65	31.6	(1.3)	26.7	(1.2)	10.3	(0.6)	9.9	(0.6)	
Education (% (SE))									
≤Elementary school	17.9	(1.2)	14.0	(0.9)	5.7	(0.5)	8.7	(0.5)	<0.001
Middle school	12.5	(1.0)	8.9	(0.9)	6.6	(0.6)	9.4	(0.7)	
High school	30.6	(1.6)	29.4	(1.4)	29.2	(1.1)	33.6	(1.1)	
≥College	39.0	(1.8)	47.7	(1.6)	58.5	(1.3)	48.3	(1.3)	
Occupation (% (SE))									
Professional and clerical	21.9	(1.5)	33.0	(1.6)	43.2	(1.3)	36.4	(1.3)	<0.001
Sales, service, agriculture, forestry, fishery, technician, and laborer	46.2	(1.6)	43.8	(1.6)	42.9	(1.3)	50.8	(1.3)	
Unemployed	31.9	(1.5)	23.2	(1.3)	13.9	(0.8)	12.9	(0.7)	
Income level (% (SE))									
Low	35.0	(1.5)	23.0	(1.3)	21.8	(1.0)	23.1	(1.0)	<0.001
Middle-low	23.7	(1.3)	27.2	(1.4)	23.6	(1.0)	25.6	(1.0)	
Middle-high	23.9	(1.3)	24.8	(1.4)	26.4	(1.0)	26.1	(1.0)	
High	17.5	(1.2)	25.0	(1.3)	28.2	(1.2)	25.2	(1.1)	
Smoking (% (SE))									
Never	32.2	(1.5)	30.5	(1.5)	22.9	(1.0)	11.4	(0.7)	<0.001
Past	45.0	(1.6)	44.6	(1.4)	43.7	(1.1)	40.4	(1.1)	
Current	22.9	(1.4)	24.9	(1.3)	33.4	(1.1)	48.2	(1.1)	
Physical activity (% (SE))									
Light	24.5	(1.4)	22.4	(1.3)	17.7	(0.9)	18.9	(0.9)	<0.001
Moderate	48.2	(1.6)	50.4	(1.5)	53.5	(1.2)	54.0	(1.1)	
Vigorous	27.3	(1.4)	27.2	(1.3)	28.8	(1.1)	27.1	(1.1)	
Sleep duration (hours, % (SE))									
<7	41.6	(1.6)	39.5	(1.5)	43.0	(1.2)	44.2	(1.1)	<0.001
7–<9	46.8	(1.7)	51.9	(1.4)	51.3	(1.2)	48.2	(1.1)	
≥9	11.6	(0.9)	8.6	(0.8)	5.7	(0.5)	7.6	(0.6)	
Total energy intake (kcal/day, mean ± SE)	2029.1	±24.6	2144.8	±23.2	2286.0	±20.0	2470.5	±20.2	<0.001

^a^ Quantity of ≥7 glasses/once. ^b^ *p*-values by Rao–Scott chi-square test for categorical variables or ANOVA for continuous variables.

**Table 4:** Nutrient intakes related to obesity according to frequency of binge drinking in Korean adult men aged ≥30 years, 2016 to 2020 Korea National Health and Nutrition Examination Survey.

	Never (*n* = 1477)		Non-Binge Drinking (*n* = 1541)		Frequency of Binge Drinking ^a^				*p*-Values ^b^
					<1 Time/Week (*n* = 2521)		≥1 Time/Week (*n* = 2641)		
Total energy intake (kcal/d)	2057.8	±23.9	2140.9	±22.4	2205.5	±19.1	2401.6	±20.2	<0.001
Fat (g/d)	44.9	±1.0	48.0	±0.9	49.9	±0.8	48.0	±0.8	0.002
Sodium (mg/d)	3779.2	±63.2	3875.1	±63.1	3993.1	±45.8	4169.8	±51.6	<0.001
Vitamin C (mg/d)	71.6	±4.7	73.6	±2.8	66.2	±1.8	62.9	±2.2	0.022

Age-adjusted mean ± SE. ^a^ Quantity of ≥7 glasses/once. ^b^ *p*-values by ANOVA for continuous variables adjusted by age.

**Table 5:** Odds ratios (ORs) and 95% confidence intervals (95% CIs) for obesity by age group, sleep duration, and alcohol intake in Korean adult men aged ≥30 years, 2016 to 2020 Korea National Health and Nutrition Examination Survey.

	Obese (n = 3467)	Controls (n = 4804)	OR ^a^ (95% CI)
Age (years)			
30–39	775	791	1.00 (reference)
40–49	821	955	1.06 (0.87–1.30)
50–64	1119	1538	1.27 (0.91–1.77)
≥65	752	1520	1.25 (0.73–2.12)
Sleep duration (hours)			
<7	1546	1886	1.28 (1.15–1.43) **
7–<9	1656	2400	1.00 (reference)
≥9	265	518	0.87 (0.69–1.10)
Frequency of binge drinking ^b^			
Never	549	928	1.00 (reference)
Non-binge drinking	568	973	0.94 (0.78–1.13)
<1 time/week	1064	1457	1.01 (0.84–1.20)
≥1 time/week	1250	1391	1.39 (1.17–1.65) **

^a^ Adjusted for age (years, continuous), education (≤elementary school, middle school, high school, or ≥college), occupation (professional and clerical, sales, service, agriculture, forestry, fishery, technician, and laborer, or unemployed), income level (low, middle-low, middle-high, or high), smoking (never, past, or current), physical activity (light, moderate, or vigorous), total energy intake (kcal/day, continuous), fat intake (g/day, continuous), sodium intake (mg/day, continuous), and vitamin C intake (mg/day, continuous). ^b^ Quantity of ≥7 glasses/once. ** *p* < 0.01.

**Table 6:** Odds ratios (ORs) and 95% confidence intervals (95% CIs) for obesity by frequency of binge drinking according to strata of age group and sleep duration in Korean adult men aged ≥30 years, 2016 to 2020 Korea National Health and Nutrition Examination Survey.

	Never		Non-Binge Drinking		Frequency of Binge Drinking ^a^			
					<1 Time/Week		≥1 Time/Week	
	No. of Obese/Controls	OR ^b^ (95% CI)	No. of Obese/Controls	OR ^b^ (95% CI)	No. of Obese/Controls	OR ^b^ (95% CI)	No. of Obese/Controls	OR ^b^ (95% CI)
Age (years)								
30–64	331/444	1.00 (reference)	381/531	0.95 (0.76–1.20)	905 /1159	1.00 (0.81–1.22)	1073 /1116	1.38 (1.13–1.69) **
≥65	218/484	1.00 (reference)	187/442	0.86 (0.65–1.14)	159/298	1.06 (0.77–1.45)	177/275	1.38 (1.02–1.88) *
Sleep duration (hours)								
<7	247/353	1.00 (reference)	233/347	0.92 (0.69–1.22)	466/600	0.99 (0.76–1.31)	580/558	1.52 (1.17–1.97) **
≥7	302/575	1.00 (reference)	335/626	0.96 (0.75–1.21)	598/857	1.03 (0.82–1.29)	670/833	1.30 (1.03–1.63) *
Sleep duration of <7 h by age (years)								
30–64	163/181	1.00 (reference)	166/216	0.84 (0.60–1.18)	414/484	0.98 (0.72–1.34)	505/455	1.45 (1.08–1.95) *
≥65	84/172	1.00 (reference)	67/131	1.31 (0.81–2.13)	52/116	0.86 (0.51–1.48)	75/103	2.07 (1.28–3.33) **
Sleep duration of ≥7 h by age (years)								
30–64	168/263	1.00 (reference)	215/315	1.06 (0.78–1.43)	491/675	1.02 (0.78–1.33)	568/661	1.32 (1.00–1.75) *
≥65	134/312	1.00 (reference)	120/311	0.70 (0.49–1.01)	107/182	1.24 (0.84–1.83)	102/172	1.09 (0.74–1.63)

^a^ Quantity of ≥7 glasses/once. ^b^ Adjusted for age (years, continuous), education (≤elementary school, middle school, high school, or ≥college), occupation (professional and clerical, sales, service, agriculture, forestry, fishery, technician, and laborer, or unemployed), income level (low, middle–low, middle–high, or high), smoking (never, past, or current), physical activity (light, moderate, or vigorous), total energy intake (kcal/day, continuous), fat intake (g/day, continuous), sodium intake (mg/day, continuous), and vitamin C intake (mg/day, continuous). ** *p* < 0.01, * *p* < 0.05.

## Data Availability

The data presented in this study are available at https://knhanes.kdca.go.kr/knhanes/main.do (accessed on 1 July 2024).
